# Enhanced Lipid Production in *Chlamydomonas reinhardtii* Caused by Severe Iron Deficiency

**DOI:** 10.3389/fpls.2021.615577

**Published:** 2021-04-13

**Authors:** Elsinraju Devadasu, Rajagopal Subramanyam

**Affiliations:** Department of Plant Sciences, School of Life Sciences, University of Hyderabad, Hyderabad, India

**Keywords:** algae, *Chlamydomonas reinhardtii*, Fourier transform infrared spectroscopy, liquid chromatography, mass spectrometer, triacylglycerols, lipids

## Abstract

Microalgae are used as a source of lipids for the production of biofuels. Most algae produce neutral lipids under stress conditions. Here, lipid accumulation by the unicellular alga *Chlamydomonas reinhardtii* was examined during cultivation under iron-limiting conditions. Severe iron stress caused the cells to accumulate a significant amount of lipid, specifically triacylglycerols (TAGs), by compromising the growth. Semi-quantitative measurements by Fourier transform infrared (FTIR) spectroscopy showed an increase in both carbohydrate and lipid content in iron-stressed *C. reinhardtii* cells compared to control. Analysis by flow cytometry and thin layer chromatography confirmed that severe iron deficiency-induced TAG accumulation to fourfold higher than in iron-replete control cells. This accumulation of TAGs was mostly degraded from chloroplast lipids accompanied by overexpression of diacylglycerol acyltransferase (DGAT2A) protein. Furthermore, liquid chromatography-mass spectrometry (LC-MS) analysis demonstrated significantly enhanced levels of C16:0, C18:2, and C18:3 fatty acids (FAs). These results indicate that iron stress triggers the rapid accumulation of TAGs in *C. reinhardtii* cells. The enhanced production of these lipids caused by the iron deficiency may contribute to the efficient production of algal biofuels if we escalate to the photobioreactor’s growth conditions.

## Introduction

Photosynthetic algae capture solar energy and store it as chemical energy in the form of starch and lipids, particularly triacylglycerols (TAGs) (Merchant et al., 2007). The transesterification of TAGs achieves biodiesel production with methanol in the presence of a suitable catalyst (e.g., H_2_SO_4_). Most microalgae accumulate increased levels of TAGs under nutrient deficiency, such as nitrogen starvation. However, stress conditions like this are also accompanied by decreased growth rates. For the economical production of biodiesel from algae, increased lipid levels must be combined with a high percentage of algal growth. Various stress conditions like nitrogen starvation (Rios et al., 2015; Lin et al., 2018), iron starvation (Gorain et al., 2013), copper stress (Hamed et al., 2017), heat stress (Ördög et al., 2016), pH (Abinandan et al., 2019), and high-light stress (Fan and Zheng, 2017) have been reported to induce lipid accumulation in microalgae effectively. The nutritional requirements for the accumulation of TAGs by *Dunaliella tertiolecta* were investigated (Chen et al., 2011).

The soluble form of iron (Fe^2+^) is very limited in soil. Its low bioavailability is a significant problem for photosynthetic organisms (Glaesener et al., 2013) because iron is essential in the biochemical pathways of plants and microalgae. In oxygenic photosynthesis, iron acts as a cofactor in all-electron transfer reactions and enhances the active photosynthetic reaction centers in algae. A lack of iron decreases photosystem (PS)II function, inter-photosystem electron transport, carbon fixation rates and ultimately affects the growth of cyanobacteria and algae (Singh et al., 2005; Devadasu et al., 2016).

Limitation of micronutrients (e.g., zinc and iron) results in the conversion of membrane lipids into individual fatty acids (FAs), which leads to the formation of lipid droplets (LDs) in *D. tertiolecta* (Chen et al., 2011). The impact of N, S, P, and Mg deficiency on microalgal metabolism has been studied in *Chlamydomonas reinhardtii* (Çakmak et al., 2014). Nitrogen starvation severely inhibits algal growth and induces TAG accumulation in *C. reinhardtii* cells (James et al., 2011). Iron stress has a wide range of effects on the endoplasmic reticulum (ER) and its membrane structures in this organism, which indicates its potential influence on TAG accumulation. Therefore, we hypothesized that two-stage iron starvation, i.e., cells initially subjected to iron deficiency are further cultured in an iron-depleted medium to cause severe iron starvation, might effectively induce lipid accumulation in *C. reinhardtii*. This so-far untested treatment regime could offer a simple way of increasing lipid content in microalgae.

The eukaryotic microalga, *C. reinhardtii*, is a model organism used extensively to study photosynthesis and primary metabolism (Merchant et al., 2007). Its genome has been sequenced; it is easy to grow in culture and has a well-characterized response to adverse environmental conditions. Therefore, this organism was selected to investigate the influence of two-stage iron starvation on TAG accumulation. We recently reported the effect of iron stress conditions on cellular biomass and the proteins involved in lipid biosynthesis in *C. reinhardtii* (Devadasu et al., 2019). In the present study, we examined the effect of two-stage iron starvation on growth rate and lipid production in this species. Cells cultivated in iron-deficient conditions were also examined for carbon partitioning into lipid and carbohydrate. Severe iron starvation conditions significantly increased the production of TAGs, which will help to make biofuel production for more efficient.

## Materials and Methods

### Strain and Growth Conditions

The wild-type *C. reinhardtii* strain CC125 was obtained from the Chlamydomonas Resource Center^1^. Cells were grown photo-heterotrophically in acid-cleaned 1-L conical flasks containing 800 mL of tris acetate-phosphate (TAP) medium supplemented with Hutner’s trace elements. Cultures were incubated at 22°C on an orbital shaker (120 rpm) with continuous illumination (50 μmol ± 5 photons m^–2^ s^–1^). The media preparation method was followed according to Yadavalli et al. (2012). We have prepared the iron limiting TAP-Fe media by omitting the required concentration of iron from Hutner’s trace elements. All culture flasks and plasticware were washed at least twice with 6 N HCl to remove the metal ions and later on washed six times with MilliQ water to remove the HCl and kept in oven at 50°C for over night. High-purity chemicals were used for preparation of iron-free stock solutions. Initially, a seed culture (30 mL) was grown in TAP medium to stationary phase, and 10 mL of culture was centrifuged (3,000 × *g* for 6 min at 25°C) to harvest the cells. The supernatant was removed. The cells washed twice with iron-free growth medium (TAP-Fe, 20 mL) before using them to inoculate TAP or TAP-Fe medium (1st generation) an initial optical density (OD_750_) of 0.2 ± 0.024. After 3 days of incubation, the iron-deficient culture was used to inoculate another iron-free medium flask to an OD_750_ of 0.2 ± 0.04 to give 2nd stage iron deficiency (severe iron deficiency), where the iron concentration is negligible. Three independent cultures of each type were propagated for each experiment (*n* = 3). The growth measurements were carried out with spectrophotometer (Perkin Elmer LS-35, United States).

### Cell Count and Dry Biomass Determination

Culture growth was monitored by counting cells using a hemocytometer. For dry cell weight (dw) measurement, the cells were collected by centrifugation at 2,000 × *g* for 5 min, washed twice with distilled water, transferred to a weighed tube, and then freeze-dried −105°C for 12 h. The tube was then weighed again, and the dry cell biomass calculated as follows:

$$\text{d}\,w=\left(w_{2}-w_{1}\right)/v$$

Where *w*_1_ is the mass of the empty tube, *w*_2_ is the mass of the tube + dried cell pellet, and *v* is the sample’s initial volume.

### Measurement of Fourier Transform Infrared Spectra

Fourier transform infrared (FTIR) spectra were recorded with a Bruker Vertex 80 V (Bruker Optik GmbH) at a spectral resolution of 4 cm^–1^. The spectra were acquired with a three-bounce diamond-attenuated total reflectance sampling accessory linked to a DTGS detector (64 scans per sample). Spectra were measured in the range from 800 to 1,800 cm^–1^ (protein amide bond I signals occur at 1,642 cm^–1^ and amide bond II signals at 1,541 cm^–1^). Single-beam spectra were obtained from the dried algal cells under a nitrogen purge. Measurements were taken using an equal dw for all samples. Background single-beam spectra were recorded from the empty ATR plate under a nitrogen purge. Baseline offset corrections were made to the absorption spectra as required using the OPUS control software (v 6.5, Bruker Optik GmbH).

### Quantification of Lipid Content

The total lipids include neutral lipids, membrane lipids, and other pigments, were quantified from all the conditions. The lipid content of dried *C. reinhardtii* cells was measured as a percentage of the dry biomass. 20 mg of crushed cell powder were suspended in 1 mL of *n*-hexane. The mixture was transferred to a 10 mL glass tube, and 3 mL of *n*-hexane and 1.5 mL of isopropanol were added before shaking the tube (225 rpm) for 24 h at room temperature. 5 mL of distilled water were added, the tube was shaken for 1 min and then centrifuged (5,000 × *g*, 5 min). The n-hexane layer containing lipids was collected in a pre-weighed microfuge tube (1.5 mL), dried overnight at room temperature and then transferred to 60°C for 1 h. The prepared lipids were then weighed and the lipid content calculated as a percentage of dw.

### Cell Staining for Lipids

Lipid droplets in *C. reinhardtii* cells were observed after staining with the lipophilic fluorescent dye Nile Red (NR) (Sigma-72485) as described previously (Moellering and Benning, 2010) with minor modifications. A 50 μg/mL stock solution of NR was prepared in methanol, and this was diluted to a final concentration of 0.5 μg/mL for staining. After adding the fluorescent dye, the cell suspension was vortexed for 30 s and incubated in darkness at room temperature for 10 min. The cells were then collected by centrifugation at 3,000 × *g* for 5 min, and unbound dye in the supernatant was removed, and cells were used for further analysis.

### Confocal and Transmission Electron Microscopy of Intracellular LDs

Fluorescence and bright-field images of LDs in *C. reinhardtii* cells were acquired using a Zeiss LSM 880 confocal microscope system (Jena, Germany). Algal culture samples (1 mL) were fixed by adding iodine solution (5 μL, 2.5 mg/mL prepared in 95% ethanol). Stained (see section “Cell Staining for Lipids”) cells were then mixed with 1% low melting-temperature agarose 1:1 (v/v) at 28°C and applied to a microscope slide. Images of agarose-embedded cells were captured using a Zeiss LSM 880 confocal microscope. Fluorescence images of the cells (λ excitation – 488 nm; λ emission scan – 560–600 nm) were recorded via a 60× oil objective at a pixel resolution of 1,024 × 1,024 in an 8-bit format. The scan and laser transmission settings remained constant for all scans.

For electron microscopic analysis, cells were resuspended in 0.1 M phosphate-buffer (pH 7.2) and cell fixation was done with 2% Glutaraldehyde (Sigma) solution for 2 h under dark and then cells were washed four times in PBS buffer each for 1 h and post-fixed in 2% aqueous Osmium Tetraoxide for 3 h, later washed with deionized distilled water for six times and dehydrated in series of ethanol solutions (30, 50, 70, 90%, and three changes of 100% for 10 min each), infiltrated and embedded in araldite resin. Further, incubated at 80°C for 72 h for complete polymerization and ultra thin sections (60 nm) were made with a glass ultramicrotome (Leica Ultra cut UCT-GA-D/E-1/100) and mounted on copper grids. The sections were stained with uranyl acetate and counter stained with Reynolds lead citrate as described albeit modifications (Bozzola and Russell, 1998). Transmission electron micrographs were captured using a Hitachi 7500 (Japan) instrument.

### Nile Red Fluorescence Assay

Triolein (Sigma T9275) was used as a TAG standard for quantification. Samples (3 × 10^6^ cells) of algal cultures grown under various growth conditions were transferred to the wells of black 96-well plates with transparent bottoms. NR solution (section “Cell Staining for Lipids”) was added to each well and mixed with the cell suspension, followed by a 20-min incubation in the dark. Fluorescence produced by the interaction of NR with cellular neutral lipids was measured using a Tecan infinite 200 microplate reader (λ excitation–486 nm; λ emission–595 nm). The chlorophyll autofluorescence signal was subtracted from each measurement. The experiment was repeated with three individual cultures (*n* = 3).

### Semi-Quantitative Analysis of TAGs by Fluorescence-Activated Cell Sorting

Relative neutral lipid levels in cells were monitored with fluorescence-activated cell sorting (FACS) analysis using a Calibur high-speed flow cytometer (BD Falcon, United States). Culture samples of 1 mL were gently mixed with NR stain (0.5 μg/mL), then held in the dark for 30 min before measuring fluorescence (λ excitation–488 nm; λ emission–545 nm). Each measurement, 10,000 events were kept as a cutoff. Chlorophyll autofluorescence was then subtracted from each reading, and mean fluorescence intensity values and images were analyzed using Flowing Software v2.5.1. Three replicates were carried out with three independent samples.

### Lipid Extraction

Total lipids were extracted from cells described by Bligh and Dyer (1959) method with minor modifications. Dried algal samples of 20 mg were resuspended in 0.8 mL of chloroform/methanol (2:1, v/v) and 100 μL of 0.9% KCl solution for extraction of neutral lipids. For extraction of polar lipids, same amount of dw was taken and resuspended with chloroform/methanol (2:1, v/v). 100 μL of 1 M of KH_2_PO_4_ was added and vortexed. Sample mixture was centrifuged at 3,000 × *g* for 5 min at RT for phase separation. The lower chloroform phase containing lipids was collected. The remaining fractions were used for further lipid extraction, and this was repeated three times; the upper layer was collected, which was repeated three times. The lower chloroform phase was pooled and dried by evaporation in a stream of N_2_, and the lipids were dissolved in 20 μL of chloroform then stored at −20°C.

### Triacylglycerol Analysis by Thin-Layer Chromatography

Twenty microliter lipid extract samples were spotted onto a thin-layer chromatography (TLC) plate (TLC Silica gel 60, F254, Merck). 10 μg of glyceryl trioleate (Sigma) were also applied as a TAG standard. Chromatography was performed using a hexane/ether/acetic acid (70:30:1, v/v/v) mixture as the solvent system. The developed TLC plates were air-dried and the separated TAGs visualized by exposure to iodine vapor at 37°C for 5 min. The plates were photographed using a digital camera, and the TAG content in each sample was quantified by gray semi-quantitative analysis using Image J (ver1.45, NIH). Polar lipids were separated by 2D TLC. The first dimension was run in the solvent system of chloroform/methanol/water (30:10:1, v/v/v) and the second dimension in chloroform/methanol/glacial acetic acid/water (35:7:6:1, v/v/v). The TLC plates were heated at 95°C to evaporate the solvent and lipids visualized by treatment with 5% phosphomolybdic acid (Sigma) in 95% ethanol. Three replicates were carried out with three independent samples.

### Immunoblotting

Total protein was extracted from approximately 3 × 10^6^ cells under severe iron starvation conditions using a buffer consisting of 0.1 M DTT, 4% SDS, 0.1 M *Tris*-HCl (pH 6.8). Extracted proteins were quantified using the Bradford method, and 5 μg samples were suspended in sample buffer, heated at 60°C for 5 min and loaded on a 15% SDS-polyacrylamide gel. After electrophoresis, the proteins were blotted onto a nitrocellulose membrane (Bio-Rad, United States) as described in Towbin et al. (1979). Equal loading was confirmed with Histone H3 antibody (Product Id: AS10 710A). The expression pattern of diacylglycerol acetyltransferase (DGAT2A) protein was determined by incubating the blot with anti-DGAT2A primary antibody (1:5,000) (Product Id: AS12 1874, Agrisera, Sweden) and then enzyme-conjugated IgG secondary antibody (1:10,000; Incell Technology). Immunoreactive bands were disclosed using the Chemidoc Touch system (Bio-Rad). Three replicates were carried out with three independent samples.

### Liquid Chromatography-Mass Spectrometry

Liquid chromatography-mass spectrometry (LC-MS) analysis of FAs was performed using a modified high-pressure liquid chromatography (HPLC) method (Jouhet et al., 2017). Total lipid extracts from cultured cells were dissolved in 100 μL of 0.1% acetonitrile. As an internal standard, C17:0 FA (Sigma) dissolved in 0.1% acetonitrile was added to all the samples to a concentration of 0.5 μM. The samples were centrifuged for 30 min at 450 × *g* at RT to remove the insoluble pellet, and then the supernatants were transferred to fresh tubes and vacuum evaporated at 45°C for 15 min. FA derivatization was performed by resuspending the samples in *n*-butanol and incubating them at 60°C for 20 min (Martin et al., 2014). After vacuum evaporation at 65°C for 15 min, the lipid samples were re-extracted into 0.1% acetonitrile: formic acid. From each sample, 10 μL were injected into the LC-MS system (Shimadzu, 8045). The following mobile phase conditions were employed: Buffer A: water: 0.1% formic acid (v/v), Buffer B: acetonitrile: 0.1% formic acid (v/v) with run time for 1 min and, oven temperature: 25°C. The organic buffer consisting of acetonitrile/0.1% formic acid (Buffer-A) and aqueous buffer composed of water with formic acid (Buffer-B) total run time for 2 min with 0.0–0.2 min flow rate was at 0.1 mL/min 20% of buffer-B. The organic phase from the 0.2 to 1 flow rate was at 0.5 mL/min with 100% organic buffer and then 1–1.5 min for 0.1 mL/min of flow rate organic buffer with 20% buffer-B. In the end, 1.5–2 min for a run with a flow rate of 0.1 mL/min buffer of 100% aqueous phase. The direct molecular masses of separated compounds were determined using a coupled Shimadzu 8045 mass spectrometer. Specific multiple reaction monitoring (MRM) scans were used to quantify each ion’s mass (Supplementary Table 1). The following conditions were employed for mass spectrometry (MS): source–ESI; nebulizing gas flow rate–2.5 L/min; heating gas flow rate–10 L/min; drying gas flow rate–10 L/min; interface temperature–300°C; DL temperature–250°C; heat block temperature–400°C.

### Statistical Analysis

All the parameters were represented as mean ± SD (*n* = 3). Three individual cultures were taken for each treatment under each biological replicate, and the final results represented the data obtained from three biological replicates. The significance of the differences between mean values of control and all iron-deficient culture was determined using Student’s *t*-test and showed *p* < 0.05 (^∗^).

## Results and Discussion

### Influence of Iron Deficiency on the Growth of *Chlamydomonas reinhardtii*

**FIGURE 1:**
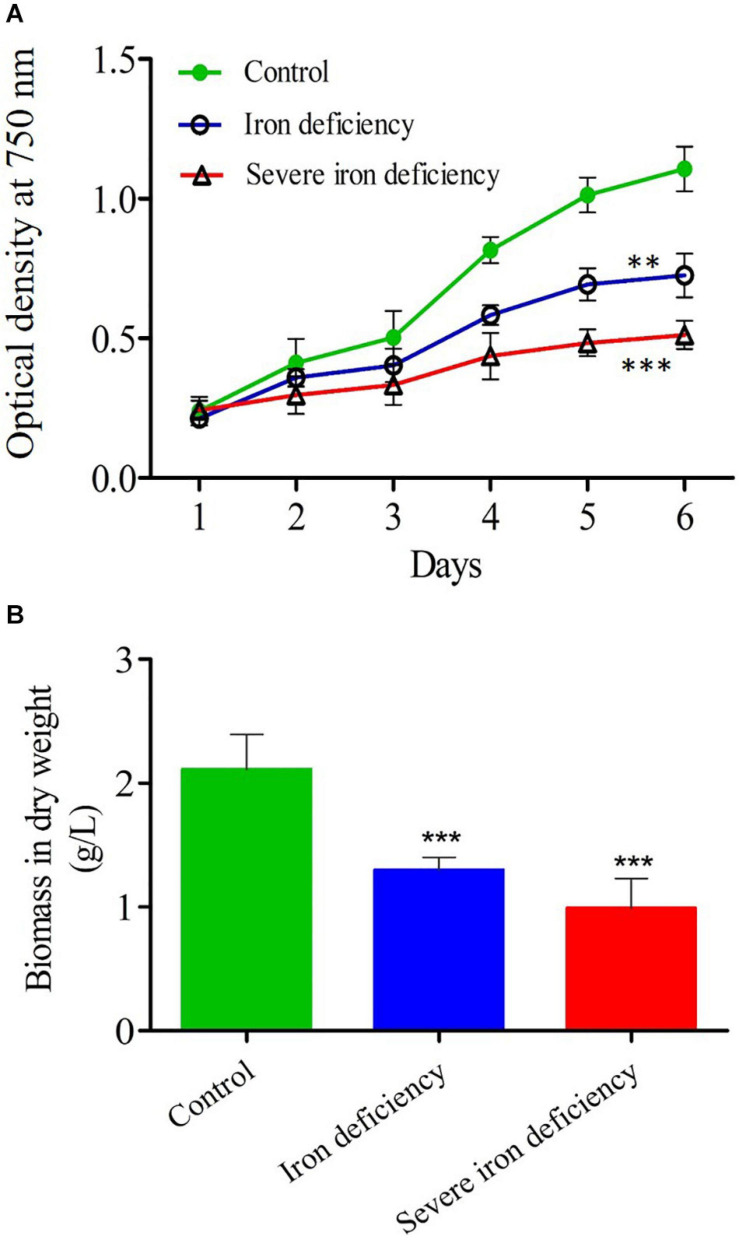
Growth and biomass measurements of *Chlamydomonas reinhardtii* cells cultured under control and iron-deficient conditions. **(A)** The optical density (OD) of control culture [20 μM iron (Fe^2+^) concentration], compared with those grown under iron deficiency and severe iron deficiency conditions. The statistical analysis was carried out from day 2 to day 6 of control and iron deficiency conditions. **(B)** Total biomass measured in the same cultures harvested after 4 days of growth. Values are mean ± SD. Statistical analysis was performed by Student *t*-test (non-paired) and *P* values were represented as **P < 0.01; ***P < 0.001. Error bar indicates the standard deviation of three biological replicates.

Tris-acetate-phosphate (TAP) medium, the standard growth medium for the culture of *C. reinhardtii*, contains 18–20 μM iron (Kropat et al., 2011). To examine the effect of iron-deficiency on growth, cells propagated in TAP were washed twice with TAP−Fe (iron-free medium) and then transferred to the same medium or TAP+Fe (iron-replete medium) as a control (Figure 1A) to give an optical density (OD_750_) of approximately 0.2. The growth of these cultures was then monitored over 4 days. In iron-sufficient conditions, the OD was doubled in 2 days and reached a maximum value (OD_750_ = 1.01 ± 0.18) after 72 h. In the inoculum, traces of iron were utilized by the cells for initial multiplication in the iron-deficient medium, so genuine iron stress only occurred after the first cell division. The OD of the iron-deprived culture decreased (OD_750_ = 0.23 ± 0.02) over the early 2 days, but it then increased over the following 4 days to a maximum value of less than half iron-replete culture (OD_750_ = 0.42 ± 0.04). Moreover, since iron is a cofactor in several enzymes such as hydrogen dehydrogenase, nitrite reductase and superoxide dismutase, normal cellular metabolism required to maintain ATP production is depressed in cells lacking iron.

Further, the iron-limited culture (1st generation) was then used to inoculate into the same iron-free medium to see further consequences (severe iron starvation) (2nd generation). In severe iron-deficient culture, the cells were wholly starved due to lack of iron in the medium. During the severe iron starvation, the cells were in the dividing stage, and the photosynthesis process was reduced significantly as they do not have sufficient iron content in the cell (Devadasu et al., 2021).

The cell growth was reduced after 4 days (OD = 0.313 ± 0.03) in severe iron deficiency. Total biomass in dw was up to 2.1 g/L in cells grown in iron-replete medium (Figure 1B). In comparison, cell biomass in the iron-limited culture was 1.0 g/L, whereas the cultures with severe iron-deficiency were further reduced to 0.45 g/L (Figure 1B). Under optimal conditions, microalgae can grow and produce higher biomass with low lipid content. In contrast, nutrient limitation can accumulate high levels of lipid content but with low biomass (Tan and Lee, 2016). A previous study on nitrogen starvation in *C. reinhardtii* showed that cell growth was severely reduced, causing a decrease in total biomass, whereas increased lipid content (Park et al., 2015). Two major obstacles to the production of biodiesel from microalgae is (1) poor growth in culture leading to low cell density with low cellular lipid content and (2) stress conditions promotes neutral lipid content while decreasing growth (James et al., 2011). Previous reports show that total lipid levels in *C. reinhardtii* cells increased dramatically by iron starvation (Urzica et al., 2013; Devadasu et al., 2019). Since continuous exposure of cells to iron limitation causes plastid and ER stress and is highly likely to result in the degradation of cells (apoptosis). It is tempting to speculate that apoptosis may be involved in this cellular degradation. This process has recently been demonstrated in Chlamydomonas, and severe ER stress leads to apoptosis in many organisms (Howell, 2013). Other reports from nitrogen and phosphate starvation lead to LDs and TAG through apoptotic mechanism (Couso et al., 2018; Masclaux-Daubresse et al., 2020) indeed, in our case, also the apoptotic mechanism would have happened. Therefore LDs and TAG could have also occurred through the apoptotic mechanism under severe iron deficiency, and the results can be seen below.

### Visualization of Intracellular LDs by Confocal and Transmission Electron Microscopy

**FIGURE 2:**
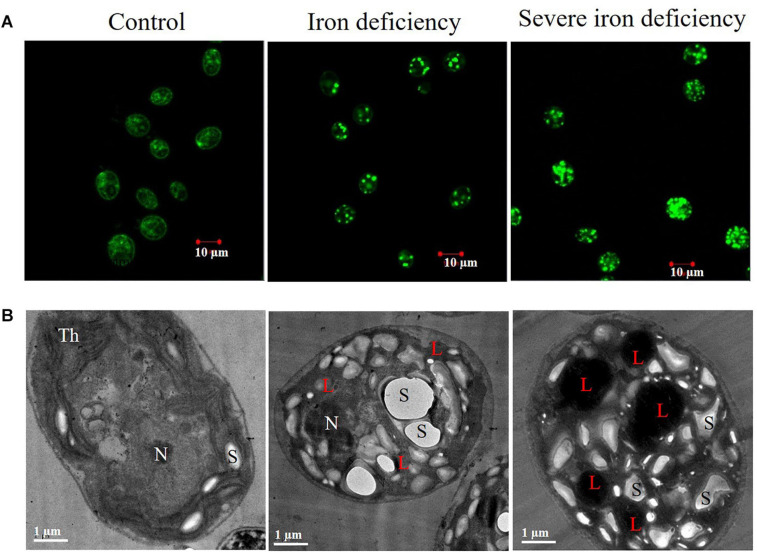
Microscopic examination is of *Chlamydomonas reinhardtii* cells cultured under iron-replete and iron-deficient conditions. **(A)** Confocal fluorescence images of cells stained with Nile Red (NR) show the location and size of lipid droplets (scale bar = 10 μm). **(B)** Transmission electron microscopy (TEM) images showed lipids accumulated within cells under iron deficiency and severe iron deficiency conditions (scale bar = 1 μm). Symbols represent, Th, thylakoids; L, lipid droplet; N, nucleus; S, starch.

The accumulation of LDs in *C. reinhardtii* cells was examined using confocal and transmission electron microscopy (TEM) (Figure 2). In iron deficiency, the size of the cells was decreased, and they displayed abnormal morphology. Cells were grown in an iron-replete (control) medium did not show any visible accumulation of lipid bodies (Figure 2A). In contrast, lipid production was induced in an iron-deficient medium, and LDs were present in cells in both iron deficiency and severe iron-limited cultures. Our study supports the earlier reports that the accumulation of LDs was seen in iron deficiency conditions (Urzica et al., 2013; Devadasu et al., 2019). Interestingly, severe iron deficiency (almost no iron in the cells or medium) caused the appearance of more abundant lipids and larger lipid bodies (Figure 2A), which has important implications for industrial applications. Previously, lipid body accumulation was observed in *Chlamydomonas* cells grown under nitrogen starvation conditions (James et al., 2011). *C. reinhardtii* cells accumulated lipids up to 45–50% of their total NR fluorescence (Moellering and Benning, 2010; James et al., 2011). In the present study, we observed lipid accumulation of 65% after 72 h of severe iron starvation compared with 45% in cells from the iron-limited 1st generation culture. This high lipid content in cells from severely iron starved cultures may make them a valuable starting material for biofuel production. TEM showed decreased unstacked thylakoid membranes in these cells. Also, the LDs observed in cells subjected to severe iron starvation (marked with “L”) were more extensive than those grown in the iron deficiency conditions (Figure 2B).

### Neutral Lipid Analysis by NR Staining

**FIGURE 3:**
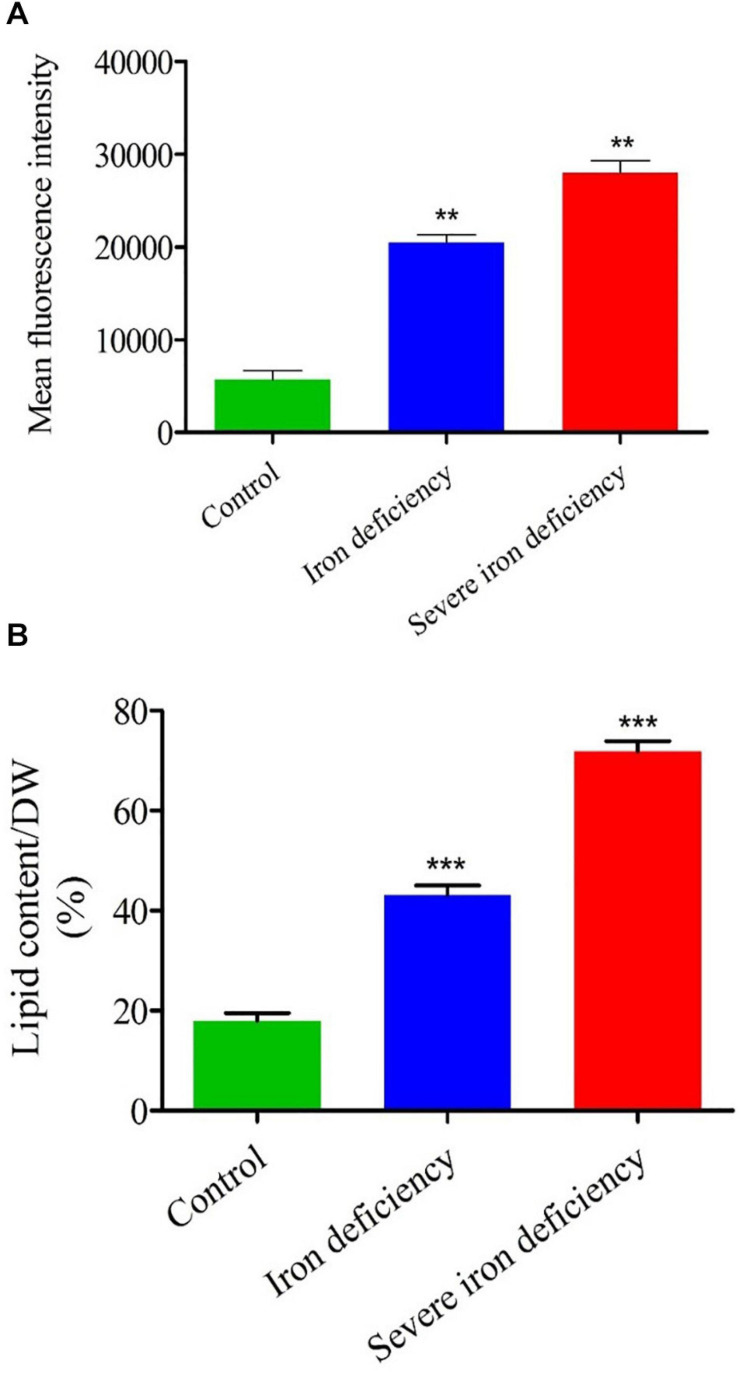
Flow cytometry (FACS) and total lipid content analysis of *Chlamydomonas reinhardtii* cells cultured under iron-replete and iron-deficient conditions. **(A)** The mean fluorescence intensity of neutral lipids stained with NR was measured by FACS. **(B)** Total lipids recovered by organic extraction were quantified in cells over 4 days of growth. Values are mean ± SD. Statistical analysis was performed by Student *t*-test (non-paired) and *P* values were represented as **P < 0.01; ***P < 0.001. Error bar indicates the standard deviation of three biological replicates.

It has been shown that nutrient limitation causes decreased cell division in microalgae, and most studied species divert FAs into TAG accumulation (Sharma et al., 2012). To examine the effect of iron deficiency on neutral lipid accumulation at the population level, 10,000 *C. reinhardtii* cells from each group (i.e., control and iron-deficient) were stained with NR after 72 h in culture, and fluorescence intensity was analyzed using flow cytometry. Many more cells with raised fluorescence levels (threefold) were present in the iron-deficient conditions. The severely iron-deprived culture showed up to sixfold fluorescence values than the control culture (Figure 3A). These fluorescence data indicate that cells subjected to severe iron deficiency are more stressed than control cells, which induces significant lipid accumulation (Figure 3A). Therefore, it is probable that the iron deficiency-induced lipid accumulation and the nitrogen starvation-induced pathway of LD formation are similar and involve the degradation and remodeling of chloroplast membrane lipids to form TAGs (Siaut et al., 2011). Notably, biomass was decreased while NR fluorescence increased when cells were grown in severe iron-depleted conditions for up to 72 h (Figure 1B). Decreased biomass and increased lipid content were observed in both iron deficiency stages, but higher levels of lipid were accumulated under severe iron starvation. The effect of iron limitation on lipid accumulation was observed from cell growth, but lipids’ accumulation was highest at fourth-day cultures (Figure 3B). These results indicate that the metabolism of iron and carbon are interlinked in *C. reinhardtii*. Neutral lipid accumulation in iron-deficient conditions occurs primarily through photosynthetic carbon fixation via the Calvin-Benson cycle (Velmurugan et al., 2014; Devadasu et al., 2019).

Neutral lipid content of cells during growth under iron-replete and -deficient conditions was monitored by staining with NR. Samples of culture were taken throughout each experiment (12–72 h), and cell densities were normalized so that an equal number of cells (3 × 10^6^) were stained (Supplementary Figure 1). As in previous studies, NR staining was employed to evaluate the neutral lipid content of *C. reinhardtii* cells and characterize the intracellular lipid bodies (James et al., 2011). Based on plate reader assay after 72 h of cell growth, the 1st and severe iron-deficient cells exhibited twofold and threefold increases in neutral lipid content, respectively, compared with the iron sufficient control cells. A previous study on *C. reinhardtii* cells cultured under N, S, and P limitation conditions also found an increased neutral lipid content (Çakmak et al., 2014). In our earlier investigation of iron limitation in *C. reinhardtii* (traces of iron still present), cells’ neutral lipid content was increased (Devadasu et al., 2019). Similarly, TAG accumulation was observed when *C. reinhardtii* cells grown in iron deficiency (Urzica et al., 2013).

The total lipid content of cells was also determined following organic extraction and the results compared with NR staining data (Figure 3B). The level of lipid accumulation caused by severe iron deficiency was higher than that resulting from the first generation of iron deficiency (Urzica et al., 2013; Devadasu et al., 2019). Therefore, severe iron starvation conditions could be a valuable means of increasing the yield of neutral lipids in microalgae, which would benefit biodiesel production.

### Lipid and Carbohydrate Measurements by FTIR

**FIGURE 4:**
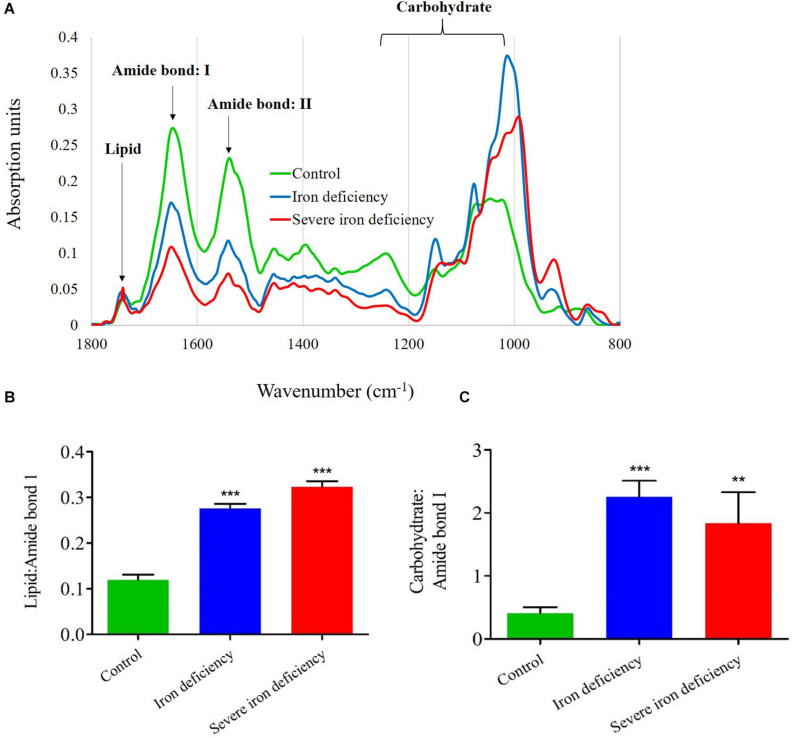
Analysis of carbon storage in *Chlamydomonas reinhardtii* cells cultured under control and iron-deficient conditions by Fourier transform infrared (FTIR). **(A)** Representative FTIR spectra of cells. **(B)** Lipid: amide I ratios over the course of 4 days in culture. **(C)** Carbohydrate: amide I ratio for the iron-deprived culture. Three independent cultures propagated in each medium were examined, and Error bars represened the means ± SD (*n* = 3). The significance values were compared with the control and they represented as ***P < 0.001 for both iron deficiency conditions in panel **(B)**; ***P < 0.001 for iron deficiency condition; **P < 0.01 value for severe iron deficiency condition in panel **(C)**.

To corroborate the NR fluorescence and total lipid data, we used FTIR to monitor carbon partitioning between lipid and carbohydrate in *C. reinhardtii* cells cultured under iron-deficient conditions (Figure 4). Based on the vibrational stretches due to peptide bonds, carbohydrates and lipid molecules, we plotted lipid ratios: amide I and carbohydrate: amide I, according to previous reports by Dean et al. (2010). Previous reports have demonstrated that microalgae are grown under N, S, and P deprivation display decreased protein content (Kilham et al., 1997; Cakmak et al., 2012). Our results also showed a drastic decline in *C. reinhardtii* cells’ protein content under 2nd stage severe iron starvation compared with 1st stage iron limitation (Figure 4). The FTIR spectra (Figure 4A) showed a strong absorption peak for the carbohydrate region (C–O–C) compared with weaker absorption for the protein amide I and amide II bands (C=O and N–H, respectively), and also for the lipid band (CO). The decrease in protein content produced by other elemental deprivations suggests that photosynthetic energy is used to synthesize more lipid and carbohydrate for storage, which correlates with our data from neutral lipid and carbohydrate measurements (Figures 3, 4). Carbon storage as lipid and carbohydrate was measured in cells cultured under all conditions after 72 h. In the 1st stage of iron-deficiency, cells showed excess storage of carbon as starch due to increased operation of the Calvin-Benson cycle of carbon fixation as previously reported (Devadasu et al., 2019). This resulted in a substantial increase in the carbohydrate: amide I ratio (fourfold) (Figure 4B). The decrease in carbohydrate content in the severe iron deficiency may be due to carbon fixation toward lipid synthesis for energy storage. Lipid content was increased in both 1st and severe iron deficiency cells after 3 days of growth: the lipid: amide I ratio increased twofold in the former while a comparatively moderate increase of threefold occurred in the latter (Figure 4C).

In contrast, cells from the severe iron deficiency culture with 1st stage of iron deficiency showed a decrease (twofold) in the carbohydrate: amide ratio after 3 days of growth compare to iron sufficient condition (control) (Figure 4C). The results of a previous study suggest that the disassociation of glycolipids [monogalactosyldiacylglycerol (MGDG) and digalactosyldiacylglycerol (DGDG)], into their individual FAs and these FAs recycling to contributes to TAG accumulation in *C. reinhardtii* (Urzica et al., 2013). Similary, in nitrogen starvation the chloroplast membrane lipids have converted to TAG accumulation in *C. reinhardtii* (Yang et al., 2018). We assume that FAs dissociated from glycolipids enter the Kennedy pathway for the synthesis of TAGs. An analysis of lipids extracted from cells from iron-deficient cultures indicated that polar lipids, especially MGDG and DGDG, are degraded more rapidly in these conditions (Supplementary Figure 2). Therefore, it is likely that these two major class of lipids of FAs are diverted to participate in the formation of TAGs.

Interestingly, the other polar lipids, DGTS and SQDG, are also decreased in severe iron deficiency, and possibly these galactolipids could have dissociated into individual FAs. A previous study on the effects of N supplementation showed growth, and it produced enhanced cell growth in microalgae and decreased intracellular lipid content (Huang et al., 2013). Iron-induced TAG accumulation was observed following the alteration of chlorophyll content, indicating that iron deficiency affects chloroplast membranes without influencing lipid accumulation. These results are corroborated by TEM images of cells showing disturbance of thylakoid stacks (Figure 2B), which indicates that membrane lipids were converted to neutral lipids. It may be concluded that FTIR is a rapid and accurate means of confirming NR staining results when determining the quality of microalgae as a biodiesel feedstock.

### Thin-Layer Chromatography Analysis of Accumulated TAGs

**FIGURE 5:**
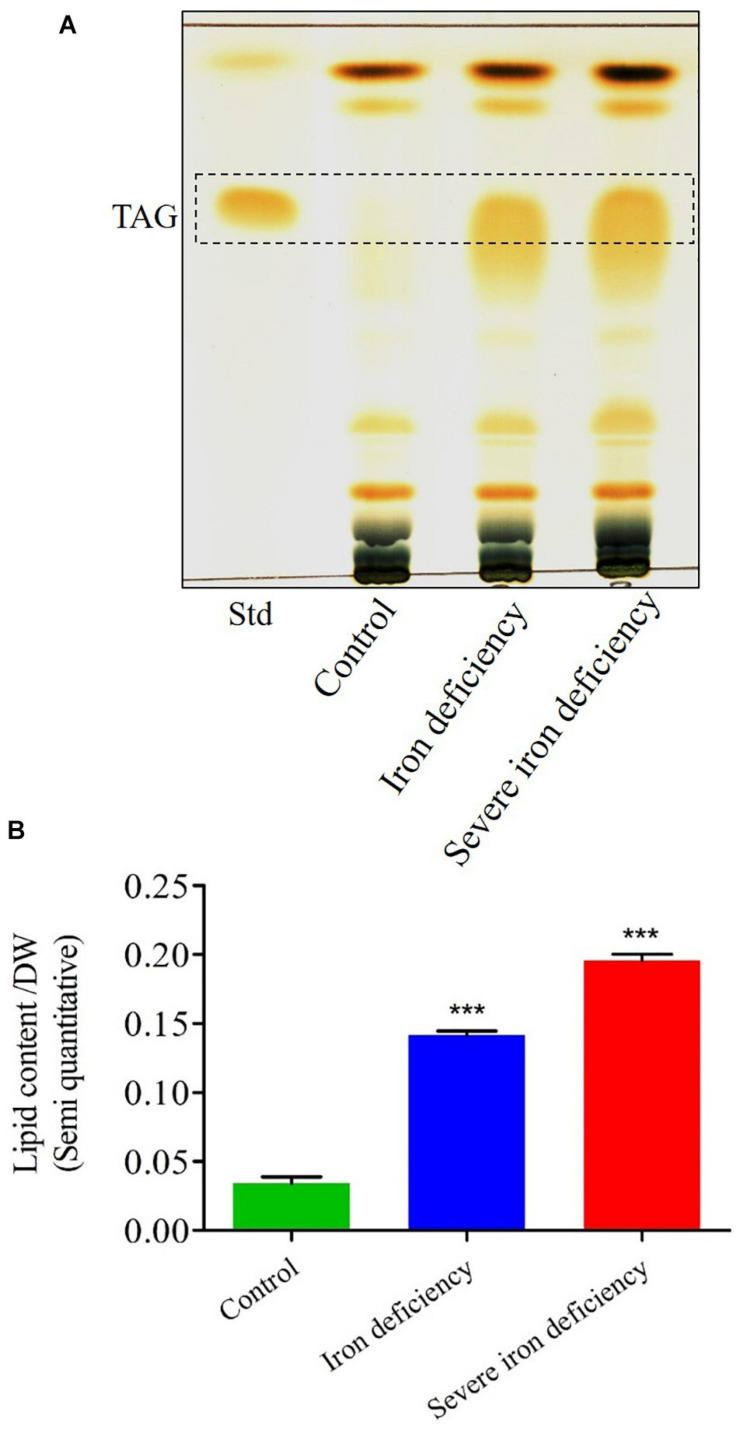
Thin-layer chromatography (TLC) analysis of total triacylglycerol (TAG) content and semi-quantitative measurement of fatty acids in *Chlamydomonas reinhardtii* cells cultured under control and iron-deficient conditions. **(A)** Lipids were extracted from equal dry cell weights (dws), and similar concentrations were loaded in each lane. **(B)** Semi-quantitative measurements of TAGs bands from the cells under control and iron-deficient conditions. Data are the mean values from three replicates (*n* = 3) expressed as a percentage of dw. The data values were done with Student’s *t*-test. Error bars represent the means ± SD (*n* = 3). ***P < 0.001.

Previous studies showed that *C. reinhardtii* gets TAGs when grown under micronutrient limitation (Deng et al., 2011; Kropat et al., 2011). Another report showed epecifically, the TAG accumulations were observed in iron deficiency (Urzica et al., 2013). However, the accumulation of TAGs in severe iron deficiency is not known so far. Following in severe iron deficiency conditions caused enhanced lipid production by *C. reinhardtii* cells under photoheterotrophic conditions, we next semi-quantified the accumulated TAGs using TLC. Total lipids were extracted from cells following growth under control, iron deficiency, and severe iron deficiency for 72 h (Figure 5). TLC analysis of these lipid extracts confirmed increased TAG accumulation during iron starvation (Figure 5A). The TAG content (semi quanitiave) of control *C. reinhardtii* cells contained 0.036 mg/g dw of lipid. In comparison, cells cultured under the 1st stage of iron deficiency showed up to lipid content (threefold) (fold increased compare to control), and 2nd stage iron deficiency had lipid contents of fourfold. These results confirmed that two-stage iron stress is a promising method for inducing lipids accumulation, specifically TAGs, in microalgae (Figure 5B). It will be necessary to determine whether this strategy can be employed in high throughput photobioreactors to generate TAGs on an industrial scale.

### Characterization of Lipids by LC-MS Analysis

Our recent report showed that *C. reinhardtii* cells subjected to 1st generation iron deficiency contain increased saturated FA levels (Devadasu et al., 2019). To determine the nature of lipids accumulation under iron deficiency conditions, we used LC-MS to characterize the FA composition. Extracted total FAs were analyzed by derivatization (transesterification), and the resultant fatty acid methyl esters (FAMEs) were analyzed by LC-MS. *C. reinhardtii* cultured under the two iron deficiency stages contained increased amounts of monounsaturated and saturated FAs compared with cells grown in iron-replete conditions (Supplementary Table 1). Levels of both FA types were higher in cells experiencing the most severe iron starvation. TAGs are rich in saturated FAs (16:0, 18:0), so their accumulation might be due to an increase in this class of FA as stated earlier (James et al., 2011; Urzica et al., 2013). A previous study demonstrated that saturated FAs form up to 20% of dw in *C. reinhardtii* cells grown under nitrogen-deficient conditions (James et al., 2011). Thus, the content of saturated FAs, which are useful for biofuels’ production, appears to be increased more by iron deficiency (Supplementary Table 1). Our analysis demonstrated that the levels of all primary FAs found in *C. reinhardtii* cells (C16:0, C16:3, C18:0, C18:3, and C18:4) are significantly increased by iron starvation. Studies on iron starvation in *C. reinhardtii* leads to the most dramatic impact on MGDG levels that decreased very rapidly due to activation of MGDG-specific lipase, which predominantly acts on newly synthesized MGDG (Li et al., 2012), which suggests that it might be involved in re-shuffling of saturated FA from MGDG to TAG accumulation. Other reports also stated that the FA desaturation mechanism is played by diiron enzymes known as FA desaturases. These FA desaturases require electrons from NADPH and reduced ferredoxin (Wada et al., 1993; Urzica et al., 2013). Thus, due to lack of iron, FA desaturases are the most sensitive targets and inhibit FA’s desaturation in *C. reinhardtii*; hence more saturated FA content was observed (Supplementary Table 1).

Moreover, other essential FAs, such as omega-3, α-linolenic acid (18:3), linoleic acid (18:2), and the omega-6 FA, oleic acid (18:1), were detected in cells subjected to iron deficiency. Alterations in FA composition have been reported previously in response to environmental changes, including temperature, pH and nitrogen deficiency in *Chlamydomonas* sp. (Poerschmann et al., 2004; James et al., 2011; Çakmak et al., 2014). The abundance of TAGs in severe iron deficiency could be an excellent strategy to boost biofuel production from green algae.

### Iron Starvation Induces DGAT2A Expression

**FIGURE 6:**
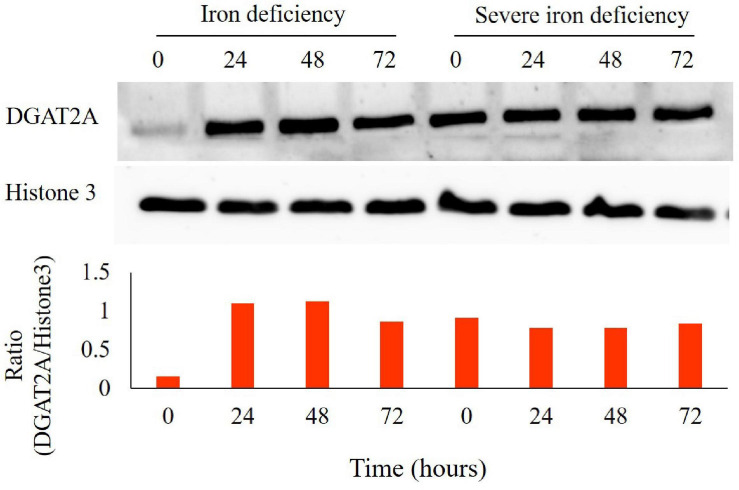
Expression analysis of diacylglycerol acyltransferase (DGAT2A) levels in *Chlamydomonas reinhardtii* cells grown in iron-deprived as well as severe iron-deficient cultures grown from 0, 24, 48, and 72 h by immunoblotting. Total proteins were extracted, and 5 μg of protein were loaded per lane after quantification. Immunostaining of Histone (H) 3 was used as a loading control (C). DGAT2A bands were quantified with Image J software. Experiments were done with three biological replicates (*n* = 3).

Triacylglycerols can also serve as storage lipids in both plants and algae. The final step in TAG biosynthesis, the incorporation of hydroxylated FAs, is catalyzed by diacylglycerol acyltransferase (DGAT) (Zhang et al., 2009). Six genes encoding this enzyme are present in Chlamydomonas, and increased expression of DGAT2A has been reported in nitrogen-starved cells (Wase et al., 2015). Previously, we observed increased DGAT2A protein level expression in *C. reinhardtii* cells during the 1st stage of iron deficiency (Devadasu et al., 2019). We also observed increased expression of DGAT2A protein level during severe iron starvation (0–72 h) (Figure 6). It is known that DGAT2A catalyzes the incorporation of diacylglycerol with FA into TAG (Zhang et al., 2009).

## Conclusion

Iron (Fe^2+^) is an essential cofactor for photosynthesis since most photosynthetic complexes contain iron. If algal cells are propagated in a medium depleted of iron, photosynthetic efficiency is reduced, but cells remain viable after 72 h in culture. In response to iron deficiency, *C. reinhardtii* cells accumulate significant amounts of lipids. Here, we observed that cells were grown in low-iron conditions accumulated lipids to levels up to fourfold higher than iron-replete controls. Transmission electron micrographs showed large LDs formed in cells subjected to the most severe iron starvation.

Furthermore, we detected a significantly increased accumulation of TAGs in severe iron deficiency about sixfold higher than the control. We also analyzed cellular FA content, which demonstrated that iron starved cells accumulated more saturated than unsaturated FAs. Notably, saturated FAs are the preferred substrate for biodiesel production.

In summary, it indicates that severe iron deficiency hampers growth and triggers the glycolipid breakdown and high TAG accumulation levels. FAs accumulated in TAG are likely derived from the various cellular metabolic networks under iron deficiency. The accumulation of TAG is high in severe iron deficiency conditions which is very important for the feedstock and biodiesel. Hence, this study could be very important for the society to improve the fishery and poultry industries using algae as a feedstock. Therefore, the photobioreactors can enhance the growth and lipid accumulation on an industrial scale that could be important in producing biofuels or aquafeed.

## Data Availability Statement

The original contributions presented in the study are included in the article/Supplementary Material, further inquiries can be directed to the corresponding author.

## Author Contributions

ED carried out the experiments, analyzed the data, and drafted the manuscript. RS designed the experiments, drafted the manuscript, and secured the fund. Both authors contributed to the article and approved the submitted version.

## Conflict of Interest

The authors declare that the research was conducted in the absence of any commercial or financial relationships that could be construed as a potential conflict of interest.

## Footnotes

1 www.chlamycollection.org

## Supplementary Material

Supplementary Figure 1 Neutral lipid content of cells during growth (12–72 h) under control and iron deficiency and severe iron deficiency conditions. Cells were stained with Nile Red (NR), and fluorescence measured using a plate reader. Three independent cultures propagated in each medium were examined. Results are the mean ± SD (n = 3).

Supplementary Figure 2 2D thin-layer chromatography (TLC) analysis of polar lipids extracted from cells grown under control, iron deficiency and severe iron deficiency conditions. Degraded lipids, monogalactosyldiacylglycerol (MGDG) and digalactosyl diacylglycerol (DGDG), phosphatidyl glycerol (PG), Sulfoquinovosyl-diacylglycerol (SQDG), and Phosphatidylinositol (PI) were detected in cells subjected to severe iron starvation. The chromatography run was repeated three times, and representative results are shown. MGDG band was quantified with ImageJ software from all the conditions and showed a percentage (%). Three independent TLC experiments were done with each culture (*n* = 3).
