# Supplementary material for: Enhanced Lipid Production in Chlamydomonas reinhardtii Caused by Severe Iron Deficiency
Source: Front Plant Sci. 2021 Apr 13;12:615577. doi: 10.3389/fpls.2021.615577 (PMC8076870; doi:10.3389/fpls.2021.615577)
Supplement: Supplementary Figure 1 — Neutral lipid content of cells during growth (12–72 h) under control and iron deficiency and severe iron deficiency conditions. Cells were stained with Nile Red (NR), and fluorescence measured using a plate reader. Three independent cultures propagated in each medium were examined. Results are the mean ± SD (n = 3). [file Data_Sheet_1.DOCX]

**SFig 1.**

Relative fluorescence intensity

Time (Hours)

**SFig 2.**

Control

Iron deficiency

Severe iron deficiency


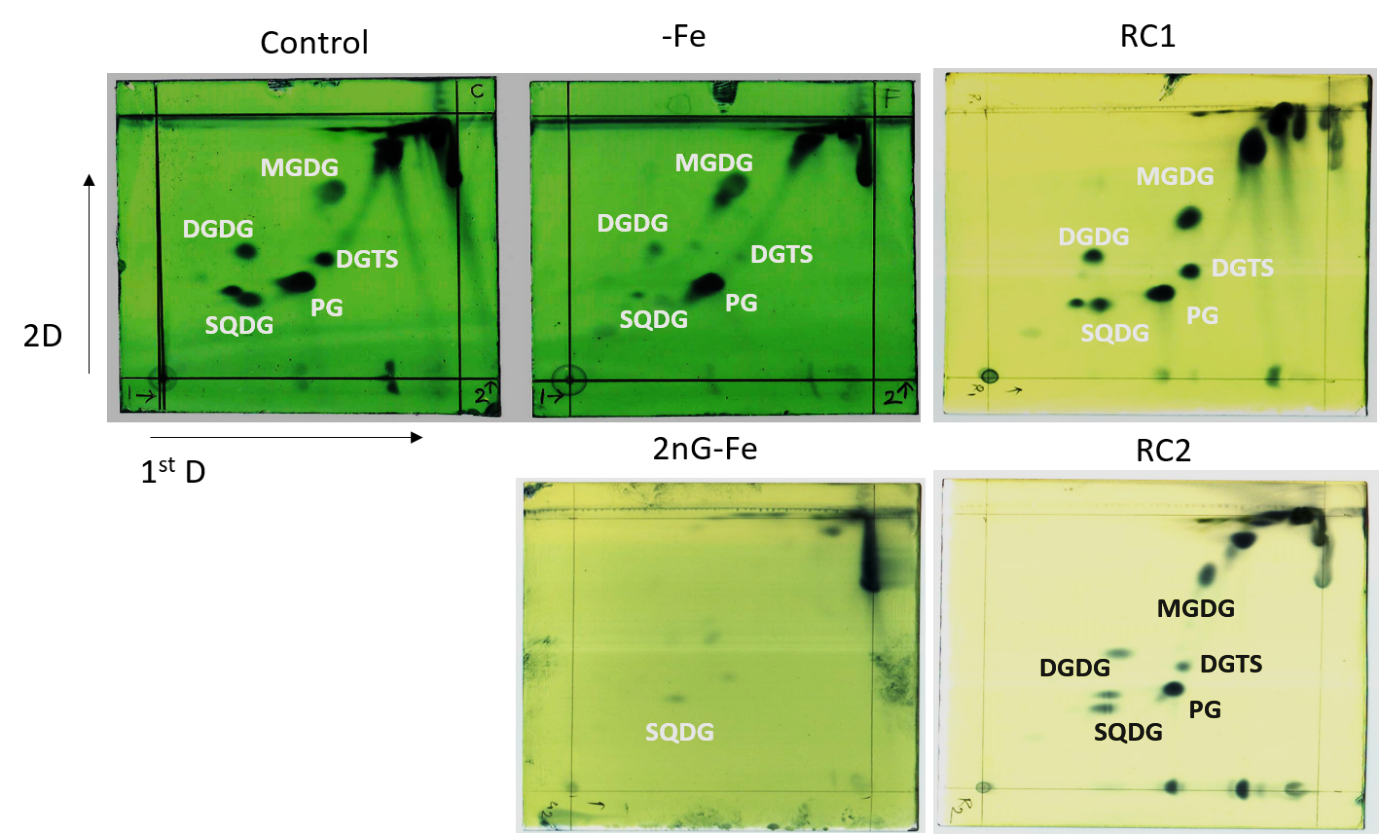


**
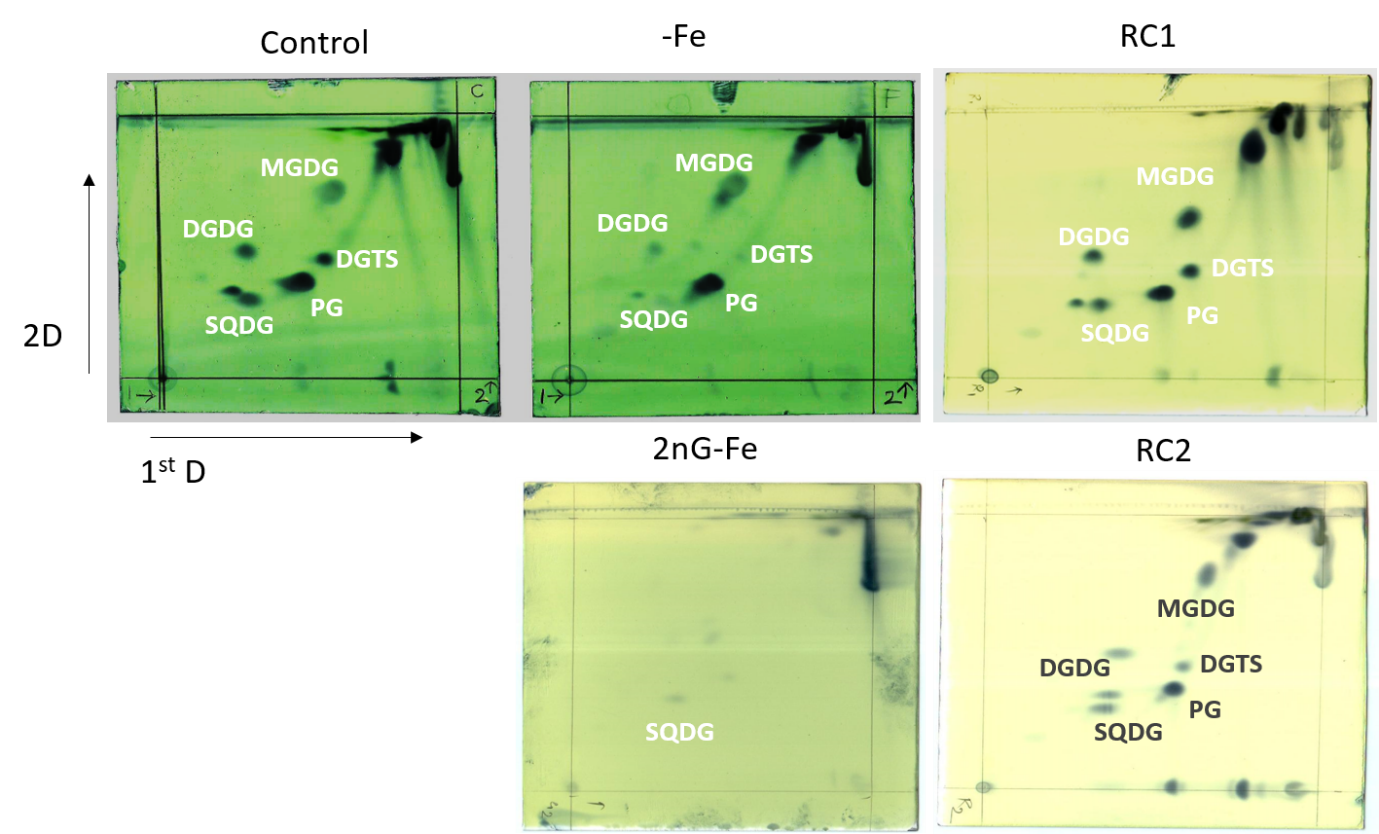
**

1D

Intensity (%)
