# Supplementary material for: Enhanced Lipid Production in Chlamydomonas reinhardtii Caused by Severe Iron Deficiency
Source: Front Plant Sci. 2021 Apr 13;12:615577. doi: 10.3389/fpls.2021.615577 (PMC8076870; doi:10.3389/fpls.2021.615577)
Supplement: Supplementary file 2 [file Table_1.docx]

**S Table 1.** The individual fatty acid identification and quantification from *C.reinhardtii* cells grown in control, iron deficiency, and severe iron deficiency conditions. Data was expressed in µmol/mg of dry weight. Mean n=3 replicates.

**FA name** **Control Iron deficiency Severe iron deficiency**

C16:0 0.61 ± 0.06 3.07 ± 1.63 3.90 ± 0.47

C16:1 1.28 ± 0.26 4.53 ± 1.15 5.55 ± 0.81

C16:2 1.94 ± 1.37 3.21 ± 0.55 3.16 ± 0.62

C16:3 1.06 ± 0.24 2.33 ± 0.77 3.18 ± 0.95

C18:0 0.53 ± 0.21 4.20 ± 0.84 5.86 ± 0.85

C18:1 2.29 ± 1.17 2.48 ± 0.43 1.96 ± 0.41

C18:2 1.59 ± 1.00 1.11 ± 0.60 1.81 ± 0.83

C18:3 1.58 ± 0.24 3.38 ± 1.09 4.01 ± 0.21

C18:4 1.95 ± 1.01 4.49 ± 1.29 5.73 ± 0.79

**Total** 12.83 27.8 35.16

**ƩSAF** 1.14 7.27 9.76

**ƩMSF** 3.57 7.01 7.51

**ƩPUFA** 6.54 11.14 13.88
